# Aerated Lobar Collapse

**DOI:** 10.5334/jbsr.3022

**Published:** 2023-01-20

**Authors:** Laura Haddad, Hanna Salame, Denis Tack

**Affiliations:** 1 Université Libre de Bruxelles, BE; 2 Centre Epicura, BE

**Keywords:** air trapping, hyperinflation, lobar collapse, bronchial obstruction, atelectasis

## Abstract

**Teaching Point:** Air trapping is a useful sign for early detection of worsening lobar collapse in the follow-up of obstructive atelectasis.

## Case History

A 40-year-old female patient with paraplegia due to a motor vehicle accident 22 years earlier was admitted to the critical care unit after a surgery for colonic volvulus. She had a persistent left lower lobe collapse over the course of several weeks as seen in the first bed-side chest radiograph (Figure 1). This radiograph showed a left main bronchus cut-off sign, suggesting an obstructive atelectasis. Interestingly, the chest radiograph on the following day (Figure 2) revealed increased lucency and inflation of the left upper lobe, thus giving the impresssion of improved ‘ventilation’ of the left upper lobe. However, the reporting radiologist draw the attention that left upper lobe hyperinflation was most likely due to bronchial obstruction from mucus plugging, and recommended endobronchial aspiration. No specific clinical action was taken. The chest radiograph on the next day (Figure 3) showed a left upper lobe collapse in addition to the existing left lower lobe collapse. A bronchoscopy was performed and a mucus plug was removed, restoring normal left upper lobe aeration seen on a chest radiograph (not shown), which was similar to the first chest radiograph shown.

**Figure:**
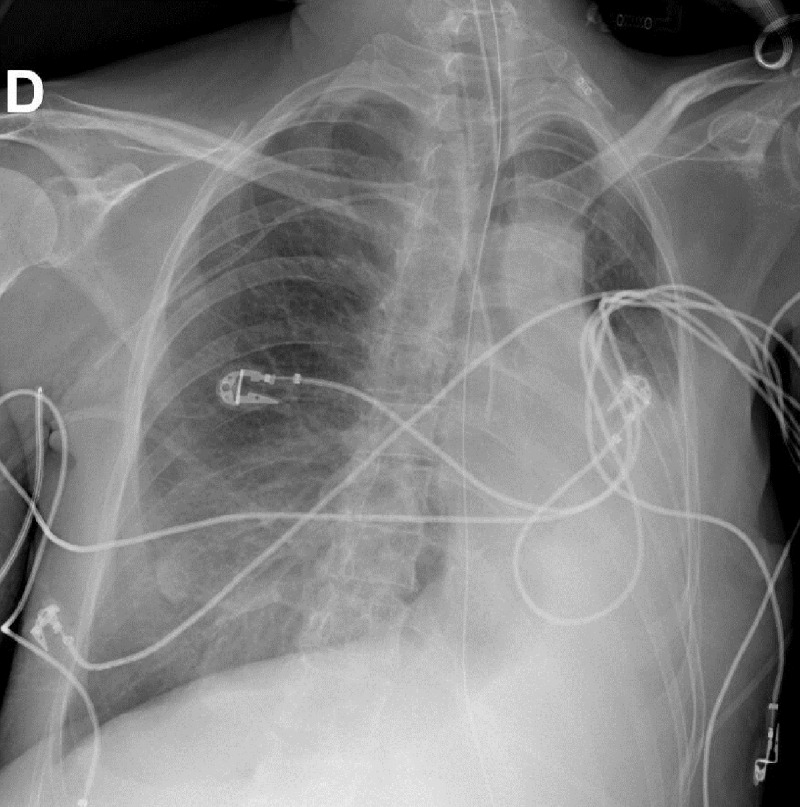
Figure 1

**Figure:**
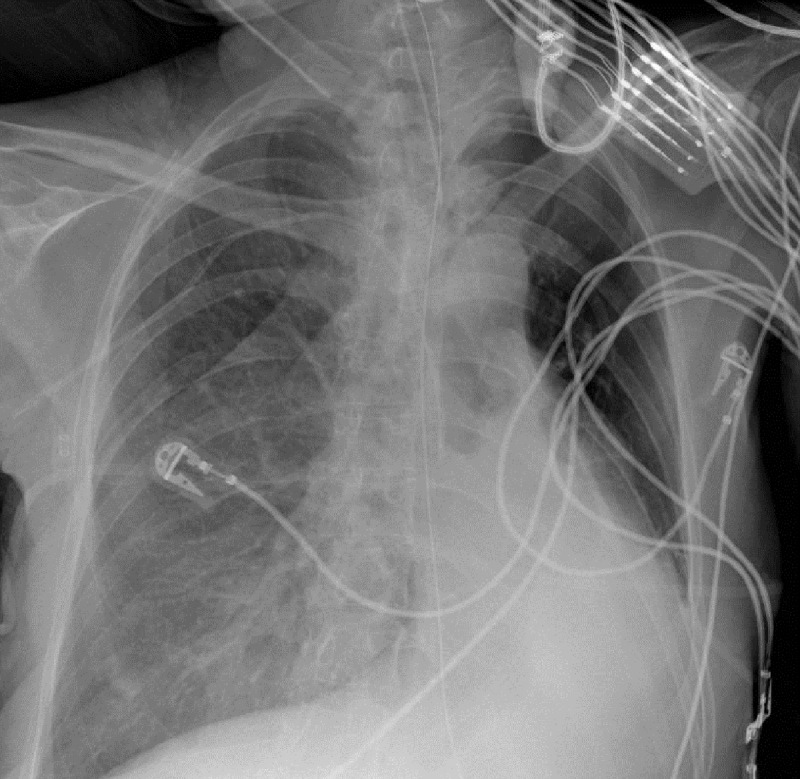
Figure 2

**Figure:**
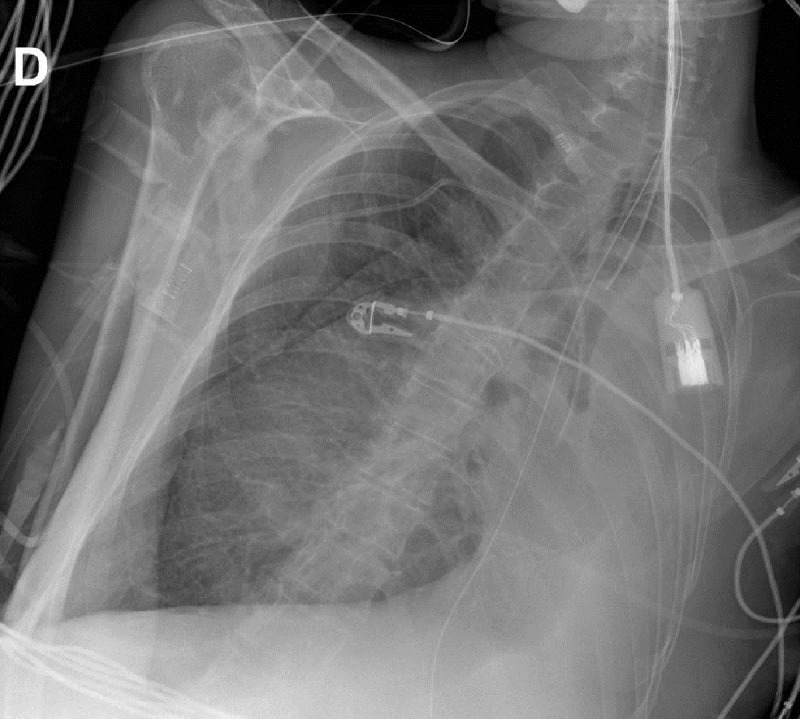
Figure 3

## Comment

Collapse (or atelectasis) is defined as the absence of gas exchange and loss of volume of a pulmonary lobe or a whole lung. Atelectasis can be of obstructive or non-obstructive etiologies. In adults, obstructive atelectasis can be caused by a tumor or a mucus plug. In case of central bronchial obstruction, collapse with loss of volume may be preceded by air trapping or hyperinflation due to a valve-like mechanism of the bronchial obstruction. The same mechanism of air trapping occurs in the setting of bronchiolitis obliterans in which the obstruction occurs at the level of small airways by a fibroproliferative process [1]. Of notice, air trapping in expiratory computed tomography (CT) can be a physiological process seen in healthy individuals [1]. This chest X-ray series offers a striking example of the brief period of hyperinflation before total collapse, when the central obstruction is complete in expiration, but not in inspiration. This hyperinflation, associated with the absence of gas exchange, should be considered as an aerated lobar collapse since the loss of volume is not yet visible. Similarly, air trapping is a commonly used feature for early detection of bronchiolitis obliterans in the setting of subclinical chronic allograft rejection [1]. Recognizing lobar or pulmonary air trapping as an abnormal finding and not a sign of improvement in the follow up of pulmonary collapse, is of important clinical relevance as it can help to advise bronchial aspiration before total collapse occurs.

## References

[1] Bankier A, Van Muylem A, Knoop C, Estenne M, et al. Bronchiolitis obliterans syndrome in heart-lung transplant recipients: Diagnosis with expiratory CT. Radiology. 2001; 218: 533–539. DOI: 10.1148/radiology.218.2.r01fe09533 11161175

